# Supplementary material for: Transcranial Direct Current Stimulation Enhances Cognitive Function in Patients with Mild Cognitive Impairment and Early/Mid Alzheimer’s Disease: A Systematic Review and Meta-Analysis
Source: Brain Sci. 2022 Apr 27;12(5):562. doi: 10.3390/brainsci12050562 (PMC9138792; doi:10.3390/brainsci12050562)
Supplement: Supplementary file 1 [file brainsci-12-00562-s001.zip › brainsci-1677067-supplementary.pdf]

Supplementary Figures

Study or Subgroup	tDCS Mean	tDCS SD	tDCS Total	sham tDCS Mean	sham tDCS SD	sham tDCS Total	Weight	Mean Difference IV, Random, 95% CI
Boggio 2012	0.1	4.313	15	0.3	4.696	15	5.8%	-0.20 [-3.43, 3.03]
Bystad 2016	0.58	1.19	12	-0.07	0.54	13	21.0%	0.65 [-0.08, 1.38]
Cotelli 2014	0.5	2.4	12	0.9	3.051	12	0.0%	-0.40 [-2.60, 1.80]
Gangemi(a) 2021	-0.3	2.568	13	-2.38	2.574	13	11.1%	2.08 [0.10, 4.06]
Gangemi(b) 2021	-0.28	2.552	9	-4.08	2.421	9	9.3%	3.80 [1.50, 6.10]
Gomes 2019	0.21	2.641	29	0	1.824	29	17.2%	0.21 [-0.96, 1.38]
Im 2019	1.1	4.133	11	-1.5	4.551	7	3.8%	2.60 [-1.56, 6.76]
Inagawa 2019	0.45	3.681	7	0.04	4.008	13	0.0%	0.41 [-3.08, 3.90]
Khedr 2014	1.65	0.96	23	1.22	0.82	11	21.9%	0.43 [-0.19, 1.05]
Khedr 2019	3.48	4.187	23	-0.61	3.172	21	9.9%	4.09 [1.91, 6.27]
Lu 2019	1.14	2.547	69	0.66	2.788	64	0.0%	0.48 [-0.43, 1.39]
**Total (95% CI)**			**135**			**118**	**100.0%**	**1.34 [0.45, 2.23]**

Heterogeneity: $Tau^2 = 0.84$; $Chi^2 = 20.45$, df = 7 (P = 0.005); $I^2 = 66\%$
Test for overall effect: Z = 2.96 (P = 0.003)

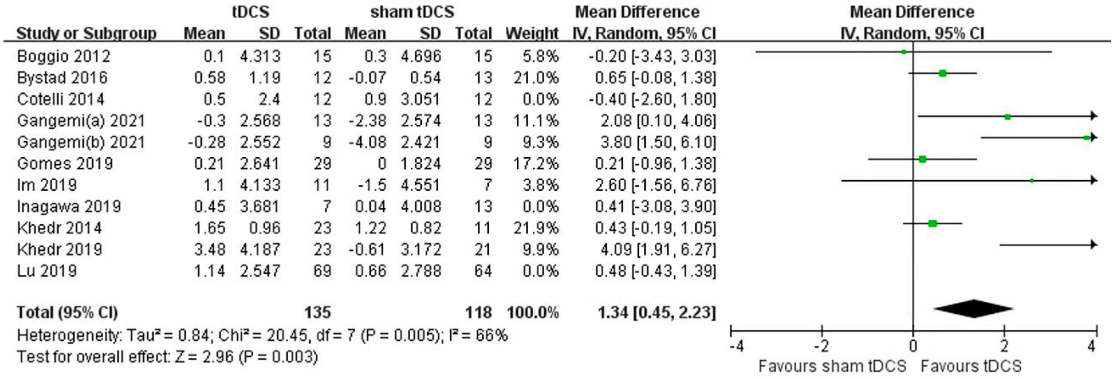

Figure.S1 Forest plots of MMSE score mean change from baseline after studies involving cognitive training were ignored.

_ES	Coef.	Std. Err.	z	P>|z|	[95% Conf. Interval]	
stimulation site	-1.759324	0.922292	-1.91	0.056	-3.566983	0.0483349
No.stimulations	1.533834	0.8543189	1.8	0.073	-0.1406007	3.208268
current density	0.0716145	0.4098593	0.17	0.861	-0.731695	0.8749239
degree of disease	-0.1302518	1.177349	-0.11	0.912	-2.437813	2.177309
_cons	0.7323193	3.304232	0.22	0.825	-5.743857	7.208496

Figure.S2 Meta-regression analyses of MMSE.

a

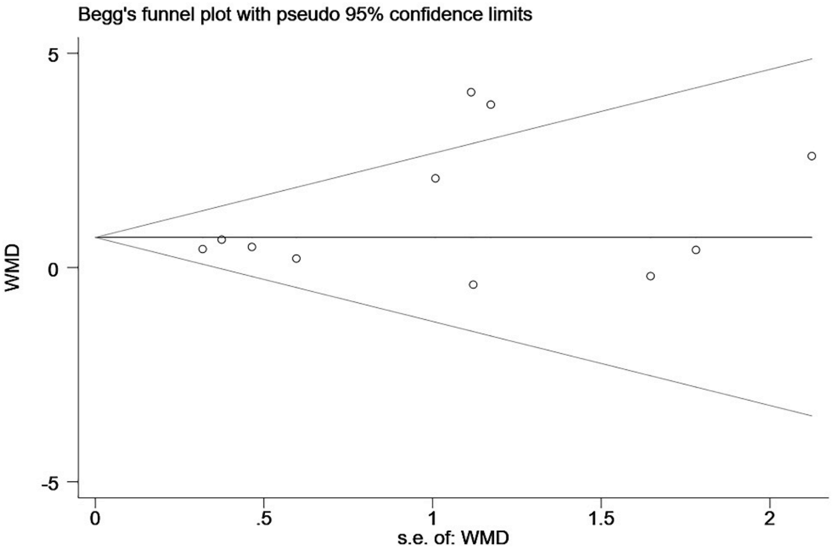

b

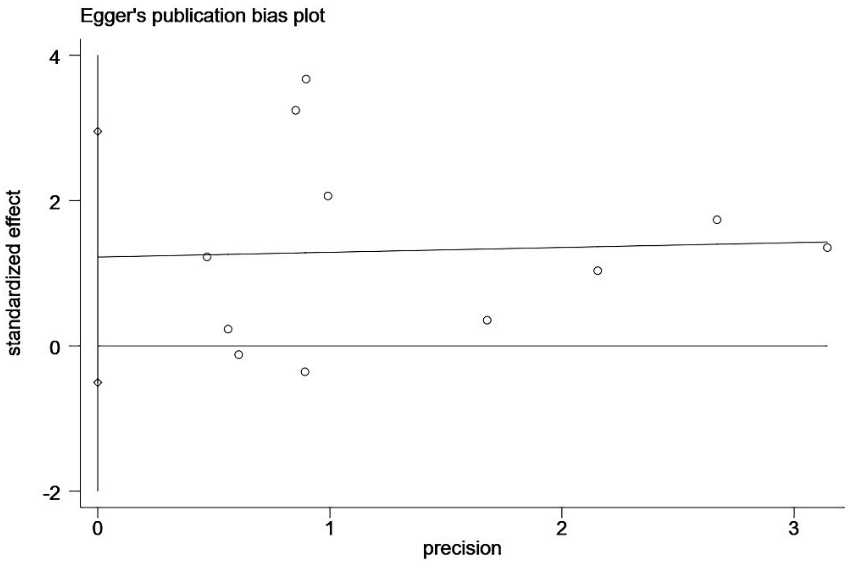

Figure.S3 Potential publication bias detection results. (a) the Begg's funnel plot, (b) the Egger's publication bias plot.
